# Three‐dimensional bladder ultrasound to measure daily urinary bladder volume in hospitalized dogs

**DOI:** 10.1111/jvim.16232

**Published:** 2021-07-31

**Authors:** Edward J. Vasquez, Allison Kendall, Sarah Musulin, Shelly L. Vaden

**Affiliations:** ^1^ Department of Clinical Sciences College of Veterinary Medicine, North Carolina State University Raleigh NC 27607 USA

**Keywords:** bladder volume, dogs, ultrasound, urine, urine residual volume

## Abstract

**Background:**

Urinary bladder volume (UBV) and urine residual volume (URV) provide important information for hospitalized dogs and might allow recognition of urine retention.

**Objective:**

Using 3‐dimensional (3D) ultrasound to monitor daily URV is a safe and effective way to recognize urinary retention.

**Animals:**

Twenty‐five client‐owned hospitalized dogs.

**Methods:**

Prospective, observational study. UBV and URV were measured using 3D ultrasound daily at approximately the same time. UBV was measured, the dog was taken for a 5‐minute controlled leash walk, then URV was estimated. Concurrent use of opioids, anesthetics, and fluids administered IV were recorded.

**Results:**

Daily URVs were >0.4 mL/kg in 22 of 25 dogs on at least 1 day of hospitalization. Seventeen of 25 dogs had an abnormal URV at the time of discharge. Of 18 dogs that were anesthetized while hospitalized, 16 had a URV >0.4 mL/kg with a mean of 4.34 mL/kg (range, 0.5‐13.4 mL/kg). No statistical difference in degree of URV was found based on the use of anesthesia, administration of fluids IV, or opioids. Weight was significantly associated with URV; dogs <10 kg had a higher URV per unit mass than dogs >10 kg (*P* = .001).

**Conclusions and Clinical Importance:**

Use of a 3D ultrasound device to measure daily UBV and URV in hospitalized dogs provides a safe estimate of bladder volume in real‐time. Monitoring daily URV might help in early identification of patients that are retaining urine, thereby preventing potential adverse effects of urethral catheterization or prolonged urinary retention.

**Abbreviations**
2D: 2‐dimensional
3D: 3‐dimensional
CAUTI: catheter‐associated urinary tract infection
CRI: constant rate infusion
NCSU‐VH: North Carolina State University Veterinary Hospital
UBV: urinary bladder volume
URV: urine residual volume
UTI: urinary tract infection

## 1 INTRODUCTION

Urine residual volume (URV) is the volume of urine remaining in the bladder after the completion of voiding and is a clinically important measurement for assessing bladder function. Using 2‐dimensional (2D) ultrasound calculations, dogs have a reported normal URV of 0.2 to 0.4 mL/kg, but with a wide range of values of 0.1 to 3.4 mL/kg.^1^ Dogs retaining >0.4 mL/kg are suspected to have incomplete voiding. Furthermore, in human medicine, a persistent elevated URV is suggestive of urine retention and can lead to serious clinical consequences such as detrusor atony and urinary tract infection (UTI).^2^, ^3^, ^4^, ^5^ Multiple factors, such as opioids, anesthesia, and surgery are implicated to cause urinary retention in people, which has led to routine in‐hospital monitoring of bladder size.^2^, ^6^ Monitoring for urinary retention in hospitalized dogs is not routinely performed and the prevalence of urinary retention in dogs is unknown.

Urine residual volume can be measured directly via urethral catheterization or indirectly via 2D ultrasound.^7^, ^8^, ^9^, ^10^, ^11^ However, these techniques impose risks such as sedation‐related adverse events or catheter‐associated UTI, and require appropriate operator skill and equipment, respectively.^12^, ^13^, ^14^, ^15^ The incidence of CAUTI in hospitalized dogs with indwelling urinary catheters or intermittent catheterization is 8%‐32%.^12^, ^13^, ^14^, ^15^ The incidence of catheter‐associated UTIs in dogs along with the daily maintenance requirements of a urethral catheter highlights the need and importance for alternative methods to monitor daily URV.^12^, ^13^, ^14^, ^15^ While 2D B‐mode ultrasound is an accurate method of measuring URV in dogs, it requires trained personnel for adequate image acquisition and dogs positioned in dorsal recumbency, which might not be suitable for critically ill hospitalized dogs.^1^, ^8^, ^16^

Application of a 3‐dimensional (3D) ultrasound device is used for point‐of‐care volumetric assessments of the human urinary bladder and has been the method of choice for monitoring urinary bladder volume (UBV) in hospitalized people.^9^, ^10^, ^11^, ^17^, ^18^ The 3D ultrasound device is intended to be used by operators with varying levels of expertise, allows for quick “bedside” measurements, and limits examination time because it reports an estimated volume in real time. Clinical application of 3D ultrasound in veterinary medicine is validated in dogs, and accurate in determining UBV and comparable to the gold standard 2D ultrasound method.^19^, ^20^ Detection of an increased URV and frequent monitoring in hospitalized dogs could be useful in identifying early signs of urinary retention. Use of 3D ultrasound could allow for easy, point‐of‐care daily monitoring without the need for urethral catheterization.

The purposes of this study are to (a) investigate for daily urinary retention in hospitalized dogs using a 3D ultrasound device by measuring URV, and (b) evaluate for any associated factors that could be contributing to increases in URV, when present. We hypothesized that the majority of hospitalized dogs would have evidence of urinary retention characterized by an increased URV as previously reported.^1^ We further hypothesized that various factors such as general anesthesia, IV fluids, or use of opioids would contribute and that those with higher body weight would have a higher URV based on previous studies.^21^

## 2 MATERIALS AND METHODS

### 2.1 Study design and animals

This prospective, observational study was performed at the North Carolina State University Veterinary Hospital (NCSU‐VH). Dogs hospitalized in any of the available housing areas in NCSU‐VH, including the general hospital ward, intermediate care ward, or the intensive care unit were recruited. If the dog met the inclusion criteria, the protocol was reviewed with the client and signed or verbal consent was obtained. The study protocol was reviewed, approved, and conducted in accordance with the North Carolina State Animal Care and Use Committee.

Inclusion criteria included hospitalization in any of the above areas for a minimum of 24 hours, ability to walk and urinate without assistance, no behavioral aggression, and no reported lower urinary tract disorders that might affect their ability to naturally void. Enrolled dogs had to weigh ≥5 kg based on increased accuracy of the device to capture bladder tracings in dogs above this weight.^19^, ^20^ Dogs were excluded if they had suspected oliguric or anuric renal failure, any history of a neurologic disorder, because of the known impact of these disorders on urine production or bladder function, or if they received an epidural during their hospitalization.

### 2.2 Data collection

Dogs were enrolled in the study from the sample of dogs that presented to the NCSU‐VH. Recorded data for each dog included: age (years), sex, breed, body weight (kg), reason for hospitalization, approximate length of hospitalization (hours), anesthetic event during hospitalization and time of anesthesia relative to enrollment, all medications administered orally or IV, and fluids administered IV including type and rate. Within 18‐24 hours after admission into the hospital and client consent obtained, an initial UBV was measured in all dogs. Within 5 minutes after, all dogs were taken outside for a controlled 5‐minute leash walk. Micturition behaviors, including the number of attempts to urinate, successful urination attempts, voiding time (seconds), and subjective assessment of urine stream strength, were observed and recorded. An attempt was recorded if the dog displayed voiding behavior such as raising of the hind limb or crouching. A successful attempt was recorded if any urine was observed after voiding behavior was noted. The stream of urine was also observed and subjectively described as either strong, intermediate or weak, and interrupted or uninterrupted. After the 5‐minute walk and natural voiding occurred, the URV was measured. UBV and URV were remeasured in all dogs once daily while hospitalized at approximately the same time per day until the date of discharge.

**FIGURE 1:**
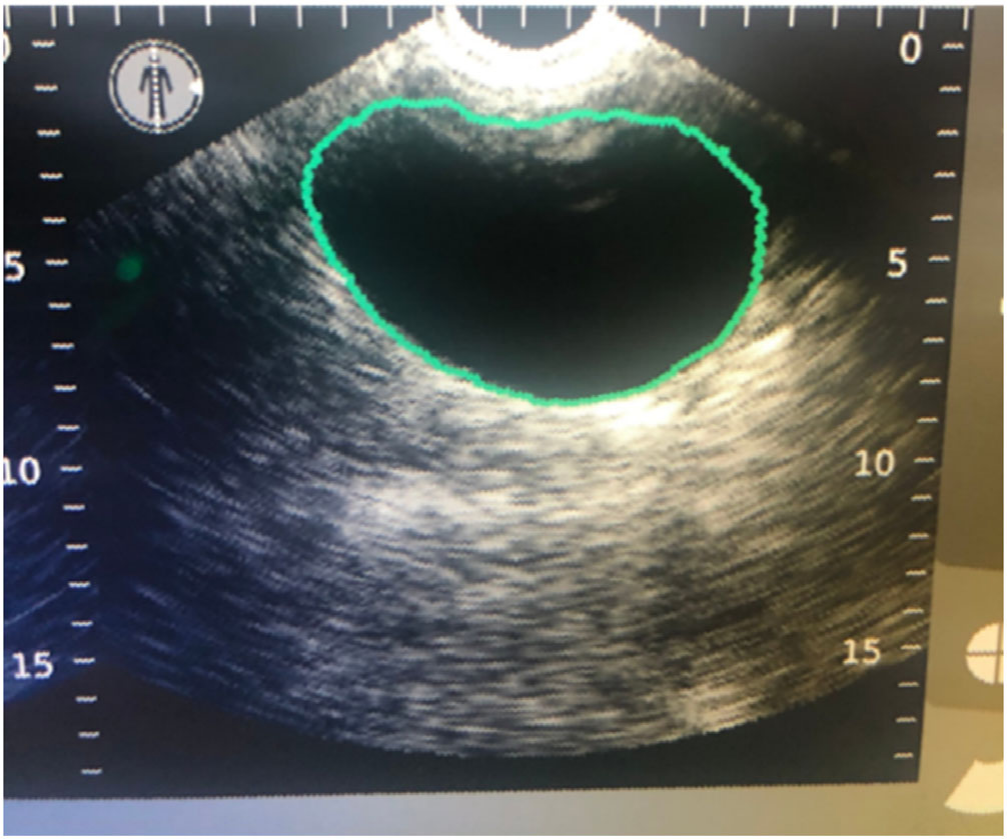
3D Verathon BladderScan Prime Plus depicting measurement of the urinary bladder. The green line represents an “acceptable” measurement of the urinary bladder volume (UBV)

Bladder volumes were measured using the 3D Verathon BladderScan Prime Plus (BladderScan Prime Plus, Verathon, Bothell, Washington) by a single operator. Dogs were placed in either right or left lateral recumbency using minimal restraint and without the need for sedation. The Verathon BladderScan Prime Plus ultrasound probe was covered in ultrasound gel and placed over the area of the bladder using minimal pressure on the skin. BladderTraq Aiming Assist provided visual clues (a green line was displayed around the image of the bladder) to indicate proper aiming (Figure 1). No probe fanning was necessary and once the bladder was in the center of the screen with a surrounding green line, a simple “point and click” technique was used. In less than 5 seconds, V_MODE_ technology automatically captured 12 B‐mode slices of the bladder and displayed the calculated volume results in real time. A total of 3 acceptable (green line) measurements were acquired for each dog, then averaged to determine the UBV and the URV. The URV was then calculated based on mL/kg.

### 2.3 Statistical analysis

All analyses were conducted in R version 3.6.3. Numerical data were assessed for normality by visual inspection. An unpaired *t* test was used to compare urinary retention rates among dogs that did or did not receive opioid therapy, anesthesia, and IV fluids. The package *lme4* was used to fit the models and *P* values were provided by the *lmerTest* package. Statistical significance was set at *P* < .05.

Because of the exploratory nature of this study and the small sample sizes, mostly descriptive rather than inferential statistics were utilized in order to eliminate type II statistical errors. Variables were described as mean ± SD. Median and ranges are also reported due to small sample size. When assessing daily URV, 3 different cutoff values were used to evaluate including 0.4, 1.0, and 3.0 mL/kg based on previous studies.^1^

## 3 RESULTS

### 3.1 Breed, age, weight, sex, hospitalization length, reason for hospitalization

Twenty‐five dogs met the inclusion criteria and were enrolled in the study. One dog represented to the hospital at a later time and was re‐enrolled for the study and measured at 2 different hospitalizations, however was counted as a single dog for the overall sample size. The demographic information for the study sample is summarized in Table 1. Reasons for hospitalization included a surgical procedure (15), supportive care (8), or radiation therapy (2). Eighteen of the 25 dogs were anesthetized during their hospitalization, occurring either daily (3), or on day 1 (5), day 2 (9), or day 3 (1) of hospitalization. Six dogs had a soft tissue surgical procedure including a total ear canal ablation (2), thoracotomy (1), splenectomy (1), forelimb amputation (1), or tail amputation (1). Four dogs had a pulmonary stenosis balloon valvuloplasty. Three dogs had orthopedic procedures including a tibial plateau leveling osteotomy implant removal (1), a tibial plateau leveling osteotomy (1), and a medial patellar luxation repair (1). Two dogs had ophthalmology procedures including a corneal graft (1) and enucleation (1). Two of the enrolled dogs underwent daily radiation therapy for metastatic anal carcinoma and mandibular mast cell tumor. The 1 dog that underwent daily radiation for metastatic anal carcinoma represented to the hospital and was re‐enrolled in the study for a second time. The remaining 8 dogs were hospitalized for supportive care for presenting complaints of: anorexia and lethargy (1), vomiting, diarrhea, and lethargy (6), and anemia and lethargy (1).

**TABLE 1:** Breed, age, body weight, sex and reason for hospitalization, and length of hospitalization of the 25 dogs enrolled in this study

Variable	Dogs
Breed (n [%])	
Labrador Retriever	6 (24.0)
Mixed breed	5 (20.0)
Miniature Schnauzer	2 (8.0)
Shih Tzu	2 (8.0)
American Bulldog	1 (4.0)
Beagle	1 (4.0)
Border Collie	1 (4.0)
Cavalier King Charles Spaniel	1 (4.0)
Nova Scotia Duck Tolling	1 (4.0)
English Bulldog	1 (4.0)
French Bulldog	1 (4.0)
Golden Retriever	1 (4.0)
Rottweiler	1 (4.0)
Shetland Sheepdog	1 (4.0)
Age (years) (mean [range])	7 (0.4‐11)
Weight (kg) (mean [range])	24.7 (5.4‐46.3)
Sex/status (n [%])	
Spayed female	12 (48.0)
Neutered male	10 (40.0)
Intact male	3 (12.0)
Reason for hospitalization (n [%])	
Surgical procedure	
Soft tissue	6 (24.0)
Cardiology	4 (16.0)
Orthopedic	3 (12.0)
Ophthalmology	2 (8.0)
Supportive care	8 (32.0)
Radiation treatment	2 (8.0)
Hospitalization length (days) (mean [range])	3 (1.5‐6 days)

### 3.2 Micturition behavior

The median number of attempts to urinate was 1 (range, 0‐4) and the median successful attempts was 1 (range, 0‐3). A total of 52 attempts were made to void, with 48 of these attempts being successful. Thirty of the 43 first attempts at voiding were strong and uninterrupted, whereas 6 of the 7 second attempts were weak and interrupted. The median observed voiding time was 13.5 seconds (range, 2‐20). Five (25%) of the dogs did not attempt to void at least once on a controlled 5‐minute walk.

### 3.3 Urinary bladder volume measurement

**FIGURE 2:**
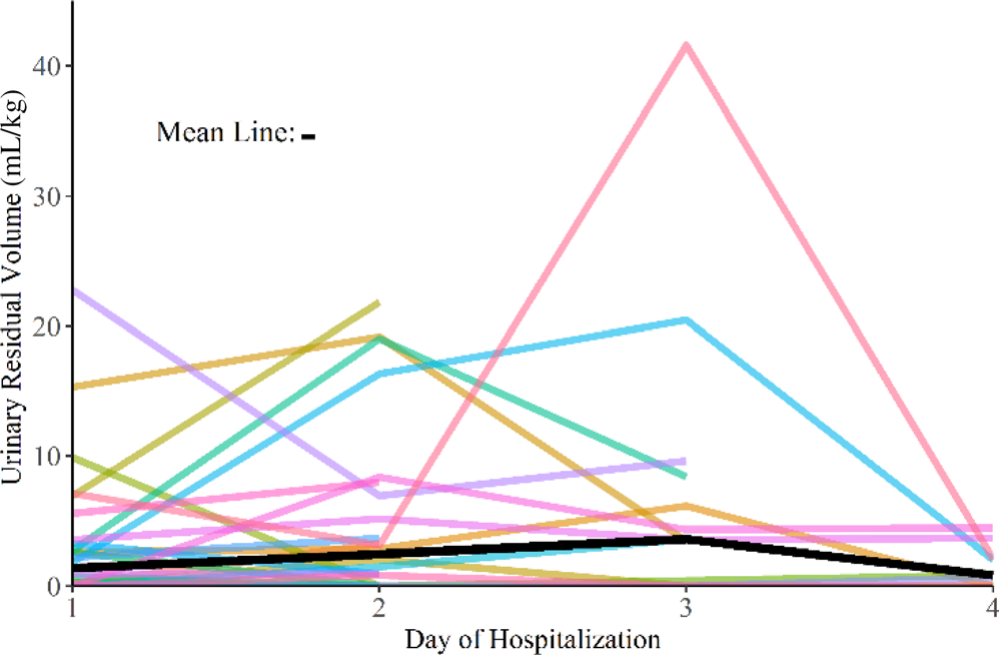
Line graph depicting daily urine residual volume (URV) in hospitalized dogs. Each line presents 1 dog in hospital

Twenty‐two dogs (85%) demonstrated a post‐void URV above the previously reported normal of 0.4 mL/kg during at least 1 time point of their hospitalization. Thirteen (50%) dogs had a URV greater than 0.4 mL/kg for every day of hospitalization (Table 2). On day 1 of hospitalization, the mean URV was 5.55 mL/kg (median, 2.98 mL/kg; range, 0.77‐22.7). Sixteen, 13, and 7 dogs had a URV above 0.4, 1.0, and 3.0 mL/kg, respectively. In the majority of dogs that remained in hospital over 60 hours (14 of 23 dogs; 61%), URV increased up until day 3, where all dogs had the greatest magnitude URV, and then started decreasing on day 4 of hospitalization (Figure 2). Seventeen dogs had a URV greater than 0.4 mL/kg on the date of discharge. Weight was significantly associated with URV, with dogs <10 kg having a higher post‐void retention per unit mass than dogs >10 kg (*P* = .001).

**TABLE 2:** Daily urine residual volume based on day of hospitalization

	Day of hospitalization					
	1	2	3	4	5	6
Retained >0.4 mL/kg?						
No^a^	10	7	3	3	0	0
Yes	16	17	10	6	1	1
Retained >1.0 mL/kg?						
No	13	10	4	5	0	0
Yes	13	14	9	4	1	1
Retained >3.0 mL/kg?						
No	18	13	4	7	1	1
Yes	7	11	9	2	0	0
Total number of dogs	25	24	13	9	1	1

^a^ No indicates that the urine residual volume for that day was below 0.4, 1.0, or 3.0 mL/kg value, whereas a Yes indicated the urine residual volume was above that value.

### 3.4 Effect of anesthesia on urinary retention

There were 28 anesthetics, 13 were daily general anesthesia for radiation therapy, and the remaining 15 were a single anesthetic procedure during their hospitalization. One dog that underwent a medial patellar luxation repair received local lidocaine (Xylocaine, Lidocaine Hydrochloride Injection, USP, Pfizer, New York, New York) at the surgical site. Three of the 5 dogs that had anesthesia on day 1 and had their first URV recorded after the anesthesia had a URV of >0.4 mL/kg (0.81, 0.94, and 5.5 mL/kg). Of these 3 dogs, 2 of these dogs had persistently increased URV on day 2 of hospitalization (0.95 and 7.97 mL/kg) and were discharged on day 2. The fourth dog was hospitalized for the next 2 days and had increasing URV from 0, 1.49, and 3.59 mL/kg on day 2 and day 3 respectively, and was discharged on day 3.

Nine dogs had anesthesia on day 2 of hospitalization after an initial measurement was obtained on day 1. Six of these dogs had URV measurements on day 1 above 0.4 mL/kg, ranging from 0.77 to 15.3 mL/kg, whereas the remaining 3 dogs did not have increased URV. Of the 6 dogs that had a URV >0.4 mL/kg on day 1, 5 had further increases in URV after their anesthesia. Three of these dogs had persistently increased URV above 0.4 mL/kg on day 3 of hospitalization. Only 1 dog that had an anesthesia on day 3 had a day 1 URV of 0 mL/kg, which increased to 8.4, 4.3, and 4.5 mL/kg on days 2, 3, and 4, respectively.

Two dogs had daily anesthesia for radiation therapy; 1 of these dogs was counted twice for total anesthesia because it represented at a later time point. All measurements were >1.0 mL/kg. The dog that represented 1 month later for another round of radiation therapy, had increased URV on day 1, suggesting that the effects of urinary retention might have persisted after the initial hospitalization.

Dogs that had anesthesia during their hospitalization did not retain significantly more than those that did not have anesthesia (mean 4.51 and 3.67 mL/kg respectively; *P* = .07).

### 3.5 Opioid administration in hospital

Twenty‐three dogs received an opioid administered IV either as part of their anesthetic protocol or for analgesia as part of their general treatment protocol. Opioids administered included methadone (Methadone Hydrochloride Injection, USP CII, Akorn Pharmaceuticals, Lake Forest, Illinois), butorphanol (Tobugesic, Zoetis, Kalamazoo, Michigan), hydromorphone (Hydromorphone Hydrochloride Injection, Hospira, Lake Forest, Illinois), and fentanyl (Fentanyl Citrate Injection, Hospira, Lake Forest, Illinois) administered as a constant rate infusion (CRI) or a transdermal fentanyl patch (Fentanyl Transdermal System, Mallinckrodt, Webster Groves, Missouri). Six dogs had bimodal pain management therapy with either fentanyl and hydromorphone (2), fentanyl and methadone (2), butorphanol and methadone (1), or butorphanol and hydromorphone (1). Three dogs received methadone alone, 4 dogs received butorphanol alone, 5 dogs received hydromorphone alone, 5 dogs were administered fentanyl as a CRI alone, whereas 4 of the 5 dogs on a fentanyl CRI transitioned to a transdermal fentanyl patch. The opioid dose was left at the discretion of the clinician of the case. Doses for fentanyl ranged from 2 to 3 mcg/kg/h as a constant rate infusion. Doses for methadone, butorphanol, and hydromorphone were noted to be 0.3‐0.5, 0.2‐0.3, and 0.05‐0.1 mg/kg, respectively.

All 23 dogs receiving opioids had a URV >0.4 mL/kg measured on at least 1 time point during hospitalization. The only time URV was <0.4 mL/kg was in 3 dogs on the day of discharge who had received a CRI of fentanyl during hospitalization. Dogs that received opioids during their hospitalization did not retain significantly more than those that did not receive opioids (mean 4.18 and 3.22 mL/kg respectively; *P* = .69).

### 3.6 Intravenous fluid therapy

Thirteen dogs received fluids IV during hospitalization, 10 started on day 1 and 3 on day 2. Fluid type and rate were at the discretion of the clinician. All dogs received a mixture of lactated Ringer's solution (lactated Ringer's Injection, USP, Hospira, Lake Forest, Illinois) and 0.45% sodium chloride solution (0.45% NaCl, Baxter Healthcare Corporation, Round Lake, Illinois). Fluid rates were left at the discretion of the clinician of the case. Fluid rates administered included: 25 mL/kg/d (1), 40 mL/kg/d (4), 45 mL/kg/d (1), 50 mL/kg/d (5), 65 mL/kg/d (1), and 90 mL/kg/d (1). Of the 13 dogs that received fluids IV, 9 dogs had a documented URV >0.4 mL/kg on day 1 of hospitalization. Only 2 of the 13 dogs that were given fluids IV did not have a URV >0.4 mL/kg. Dogs that received fluids IV did not retain significantly more over the course of their stay than those that did not receive fluids IV (mean 4.26 and 6.25 mL/kg respectively; *P* = .32).

### 3.7 Length of hospitalization

The median length of hospitalization was 3 days (range, 1.5‐6). Regardless of the cutoff value used to define urinary retention (0.4, 1.0, or 3.0 mL/kg), there was no direct effect on length of hospitalization and increasing URV (*P* = .93).

## 4 DISCUSSION

The use of 3D ultrasound has been shown as a safe, efficient, and reliable tool for measuring canine UBV, thus allowing for daily monitoring of URV as an indicator of urinary retention.^19^, ^20^ In this study, we demonstrated that hospitalized dogs had estimated URVs above reported normal cutoff values, consistent with urinary retention. Approximately 88% of dogs had a URV above 0.4 mL/kg during at least 1 time point of hospitalization, whereas 52% had a URV above 0.4 mL/kg during the entire length of hospitalization. The results of this study suggest that dogs in hospital might retain, however it is difficult to state if this elevated URV could lead to future complications.

Hospitalized dogs in the present study had URV's that peaked on day 3 of hospitalization. Multiple factors could have contributed to the increase in URV during hospitalization, including opioid administration with or without an episode of anesthesia including doses and types of opioids used, IV fluid therapy including fluid type and rate of fluid administration, and the unfamiliarity of a new environment. No significant statistical differences were noted for opioid administration, anesthesia, or administration of IV fluids; however, it is possible that the sample size was too small to detect effects from these variables, particularly when some dogs might have had multiple contributing factors.

In this study, the majority of hospitalized dogs receiving opioids had a URV >0.4 mL/kg at least once during hospitalization. This was not statistically significant when compared to dogs that did not receive an opioid; however, treatment groups were small, uneven and most dogs receiving opioids were also receiving other drugs. Opioid use has been suggested to be a risk factor for urinary retention in people; however, data on veterinary patients are limited.^22^, ^23^ A human study revealed that systemic opioid administration impairs perception of bladder fullness and the urge to urinate.^3^ Furthermore, a meta‐analysis of human medical literature revealed that urine retention is directly related to the dose of opioid used during the postoperative period.^2^ A controlled study directly comparing URVs of dogs in hospital with and without administration of opioids by different routes (ie, IV and epidural) and dose ranges in hospital is needed.

Over half of the dogs in this study were given variable rates of fluids IV starting on either day 1 or day 2 of hospitalization and all but 2 dogs had a URV >0.4 mL/kg; however, a significant difference was not detected when compared to dogs that did not receive fluids IV. In human medicine, excessive infusion of fluids IV can lead to overdistension of the urinary bladder, which can inhibit detrusor muscle function and lead to increased URV.^2^ This could be compounded in dogs that are given too few opportunities to urinate. Ultimately, study design including the administration of variable fluid rates, the administration of variable opioids at variable doses, and small group of dogs makes it difficult to isolate the effect of each of these factors on the URV.

The majority of dogs in this study underwent anesthesia during hospitalization. Although anesthesia could have contributed to urinary retention in the dogs of this study, it is difficult to assess the impact because of multiple confounding factors. Postoperative urinary retention (POUR) is well documented in human medicine.^2^, ^3^, ^4^, ^5^, ^6^, ^24^ Preoperative (age, sex,), intraoperative (IV fluids, local anesthetics, opioids, and surgery duration), and postoperative (bladder volume, sedatives, length of hospitalization, and opioids) variables are identified risk factors for POUR.^2^, ^5^, ^6^, ^24^ Furthermore, human nursing guidelines are aimed to help identify patients who might be retaining urine through routine monitoring of UBVs.^24^ No guidelines exist for veterinary patients. The majority of dogs in this study were hospitalized >60 hours and had URVs >0.4 mL/kg; however, because of the confounding variables it is difficult to ascertain the effect of length of hospitalization on urinary retention.

Contrary to previous studies which found that those of higher body weights had higher URV,^21^ we found that heavier dogs had smaller URV. These differences could be artifacts of the small sample size used in each study. Another speculative theory is that larger breed dogs could have less in‐hospital anxiety than smaller breed dogs. Behavioral traits, though not evaluated in this study, might impact micturition behavior; this potential association would be interesting to study further. Micturition behaviors were subjectively evaluated and observed during hospitalization in this study. We found that male dogs made more urination attempts than did female dogs but there was no difference in URV between male and female dogs. A previous study also found that normal male dogs made more urination attempts than did normal female dogs.^1^ The importance of this on URV is not known.

A major limitation of this study was the majority of dogs enrolled received an opioid along with fluids IV and multiple other medications including sedatives. This made it difficult to determine the effect of these factors on URV. This was an explorative study to determine if URV increases in hospitalized dogs and further investigation with a larger group and a control group is needed to further assess the effects of these variables on URV.

In conclusion, this study suggests that hospitalized dogs have URV's >0.4 mL/kg and might experience urinary retention during hospitalization; however, the long‐standing effects of this elevated URV are unknown at this time. The use of a safe and efficient “cage‐side” 3D ultrasound device to measure daily UBV and URV in hospitalized dogs provides a quick estimate of bladder volume in real‐time and decreases the need for urethral catheterization. The device could help in early identification of dogs that are retaining urine and ultimately prevent the possible complications of urinary retention.

## CONFLICT OF INTEREST DECLARATION

Shelly Vaden serves as Associate Editor for the *Journal of Veterinary Internal Medicine*. She was not involved in review of this manuscript. No other authors have a conflict of interest.

## OFF‐LABEL ANTIMICROBIAL DECLARATION

Authors declare no off‐label use of antimicrobials.

## INSTITUTIONAL ANIMAL CARE AND USE COMMITTEE (IACUC) OR OTHER APPROVAL DECLARATION

Approved by the North Carolina State Animal Care and Use Committee (protocol 19‐656‐0).

## HUMAN ETHICS APPROVAL DECLARATION

Authors declare human ethics approval was not needed for this study.
